# A Generative Framework for Predicting Antiferromagnets

**DOI:** 10.1002/advs.202509488

**Published:** 2025-09-26

**Authors:** Jianhu Gong, Zhengming Zhang, Zhenyu Fan, Hanghang Fu, Hongchang Wang, Dunhui Wang

**Affiliations:** ^1^ Zhejiang Provincial Key Laboratory of Data Storage Hangzhou Dianzi University Hangzhou Zhejiang 310018 China; ^2^ National Laboratory of Solid State Microstructures Nanjing University Nanjing Jiangsu 210093 China

**Keywords:** antiferromagnets, crystal structure prediction, crystal diffusion variational autoencoder, density functional theory

## Abstract

Predicting antiferromagnets (AFMs) is crucial for advancing ultrafast spintronics. However, traditional methods are constrained by the complexity of correlated electrons and magnetic ordering, limiting systematic exploration of new chemical spaces. Herein, an AFM design framework integrating a crystal diffusion variational autoencoder is presented with data augmentation (CDVAE‐DA), crystal graph convolutional neural networks (CGCNNs), a genetic algorithm (GA), and density functional theory (DFT) validation. CDVAE‐DA generates chemically diverse and structurally valid candidates, achieving a composition validity rate of 90.68%. Leveraging transfer learning on an AFM dataset, the CDVAE‐DA is biased toward generating AFM structures. The CGCNNs efficiently screen for potential AFMs using three properties: formation energy (FE), total magnetic moment (TMM), and band gap (BG). The GA regulates the direction of structure generation using these properties, while DFT calculations rigorously validate the lattice stability and AFM order of generated structures. Employing this framework with GA yields three AFM semiconductors (MnS, FeO_4_P, and MnO) from 2000 generated structures. In contrast, omitting GA identifies two metallic AFMs (LiVO_2_, LiFeN) from 5000 structures. This underscores the GA's role in optimizing latent vectors to facilitate efficient AFM generation. This work establishes a design paradigm for AFMs, accelerating next‐generation spintronic material discovery.

## Graphical abstract

This work establishes a design paradigm for antiferromagnets (AFM), accelerating next‐generation spintronic material discovery. By integrating a crystal diffusion variational autoencoder with data augmentation, crystal graph convolutional neural networks, and a genetic algorithm, latent vectors are updated toward AFM property spaces, expanding systematic exploration of chemical spaces.

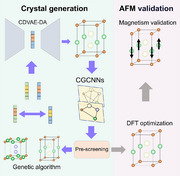

## 1 Introduction

Antiferromagnets (AFMs), characterized by the antiparallel alignment of atomic magnetic moments yielding zero net magnetization, are highly promising for next‐generation ultrafast spintronics.^[^ ^1^, ^2^ ^]^ This unique magnetic structure confers significant advantages: robustness against external magnetic fields, absence of stray fields, and intrinsic terahertz spin dynamics.^[^ ^3^, ^4^, ^5^ ^]^ Nevertheless, discovering these materials remains challenging. While high‐throughput density functional theory (DFT) has accelerated discoveries in domains like stable surfaces^[^ ^6^ ^]^ and electrodes,^[^ ^7^ ^]^ it struggles with the dual complexities of correlated electron systems and intricate magnetic ordering. This is particularly challenging for metallic magnets, where the coexistence of wide and narrow bands often drives collective electron behavior, hindering accurate description.^[^ ^8^ ^]^ Moreover, beyond‐DFT approaches employing hybrid functionals suffer severe inefficiency for metallic systems.^[^ ^9^ ^]^ Even machine learning‐enhanced workflows encounter difficulties during iterative structure relaxation,^[^ ^10^ ^]^ slowing progress in AFM development. Crucially, existing methods fail to systematically probe uncharted chemical spaces beyond known structural prototypes.^[^ ^11^, ^12^, ^13^ ^]^

It is well established that material properties are intrinsically determined by their crystal structure, and traditional approaches predominantly focus on predicting properties based on known structures. Conversely, inverse materials design entails the prediction and design of microstructures guided by predefined target property ranges.^[^ ^14^ ^]^ This approach not only significantly shortens the discovery cycle but also dramatically expands the explorable chemical spaces.^[^ ^15^ ^]^ Recent advances in deep generative models present new opportunities for crystal structure prediction through data‐driven distribution learning. The crystal diffusion variational autoencoder (CDVAE) generates diverse crystal structures with chemically valid compositions, moderate bond lengths, and thermodynamic stability,^[^ ^16^ ^]^ demonstrating strong applicability in predicting structures exhibiting ultrahigh lattice thermal conductivity^[^ ^17^ ^]^ and designing catalyst surfaces.^[^ ^13^ ^]^ Concurrently, the MatterGen model employs equivariant score networks during the reverse diffusion process to achieve stepwise restoration of atomic coordinates, species, and lattice parameters, thereby enabling the generation of crystal structures with targeted crystallographic symmetries.^[^ ^18^ ^]^ However, generative models face several persistent challenges. First, networks trained directly on sparse material datasets with target properties typically suffer from compromised generalization capabilities, hindering high‐performance predictive model development. Second, the incorporation of AFM ordering constraints remains unaddressed in current frameworks. Third, these models inherently exhibit distributional bias toward training‐data‐like outputs, fundamentally limiting exploration beyond established chemical spaces and constraining the discovery of novel functional materials.^[^ ^13^ ^]^

In machine learning, data augmentation (DA) techniques such as input rotation enhance training diversity, enabling networks to effectively capture essential data features.^[^ ^19^, ^20^ ^]^ Concurrently, transfer learning leverages pre‐training on large datasets to achieve robust predictive accuracy even with limited target‐domain data.^[^ ^21^, ^22^, ^23^ ^]^ For crystal property screening, the crystal graph convolutional neural network (CGCNN) provides efficient evaluation of structure‐property relationships.^[^ ^24^, ^25^ ^]^ Crucially, by processing magnetic moments with crystal datasets, CGCNN would demonstrate significant potential for identifying fundamental AFM characteristics. Most pivotally, a genetic algorithm (GA)^[^ ^26^, ^27^ ^]^ iteratively optimizes latent vectors toward target property preferences, thereby enabling expansion of explorable chemical spaces.

In this work, we introduce an integrated framework for AFM discovery that synergistically combines CDVAE with DA (CDVAE‐DA), CGCNN, GA, and DFT validation. The workflow proceeds in four steps: i) CDVAE‐DA generates chemically diverse candidates through transfer learning on MP‐20 and AFM‐specific datasets; ii) CGCNNs screen for AFM candidates based on the following criteria: formation energy (FE) <0 eV atom^−1^, band gap (BG) between 0 and 1 eV, and total magnetic moment (TMM) in the range of 0–0.7 *μ*_B_ f.u.^−1^; iii) GA updates latent vectors toward target property spaces; iv) DFT performs relaxation and verifies AFM stability. Implementation with GA yields three AFM semiconductors (MnS, FeO_4_P, MnO) from 2000 generated structures, whereas exclusion of GA identifies two metallic AFMs (LiVO_2_, LiFeN) from 5000 structures. This demonstrates the GA's efficacy in optimizing latent vectors for efficient AFM generation. Collectively, our work establishes a generative paradigm that bridges crystallographic constraints with AFM ordering prediction, accelerating the discovery of next‐generation spintronic materials.

## 2 Strategy

### 2.1 Design Framework of the AFMs

**Figure 1:**
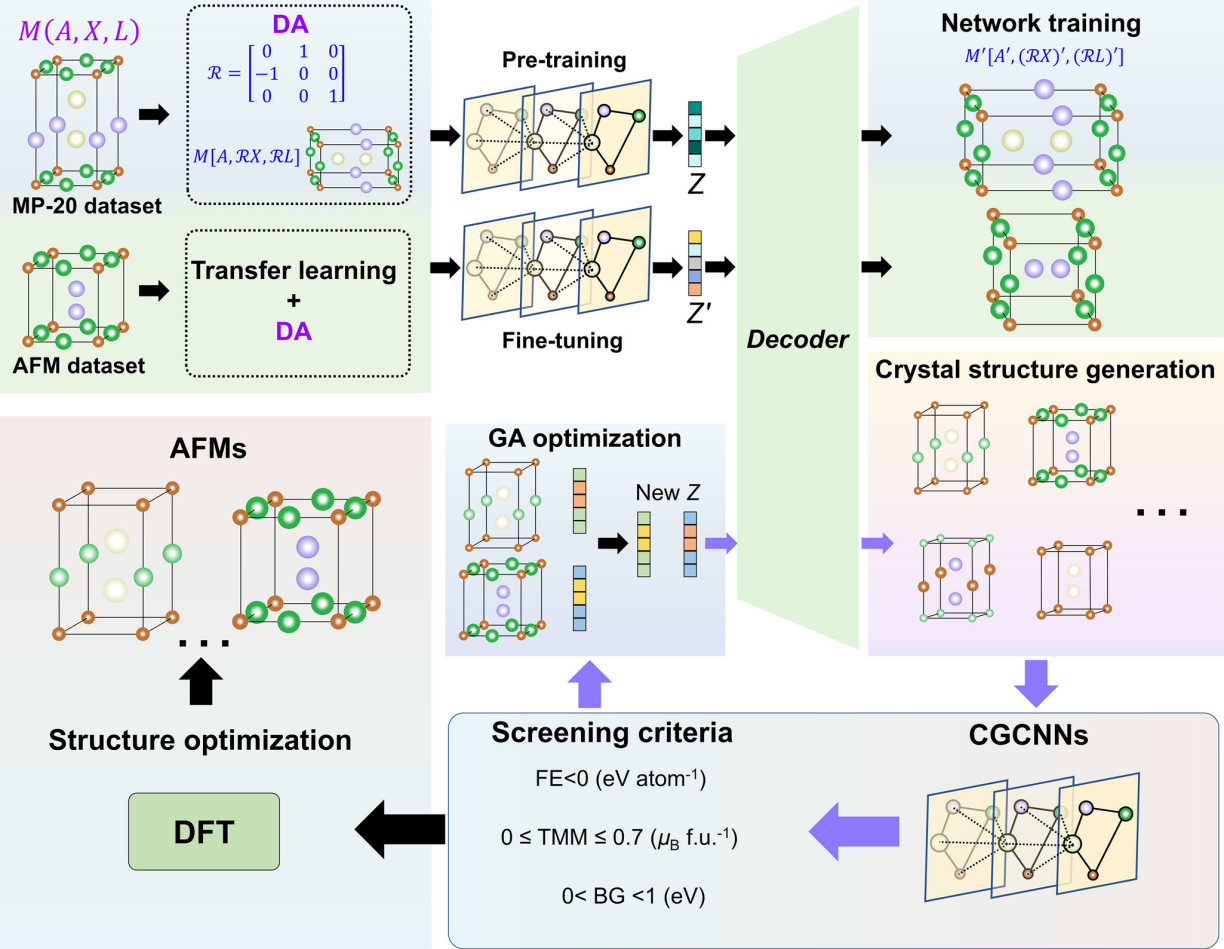
Design framework for discovering AFMs. Crystal structures are input to the CDVAE‐DA. DA applies affine rotations R to reorient lattice vectors and coordinates into specific orientations per training batch. Transfer learning is utilized to assist the CDVAE‐DA in training on limited AFM datasets. Three structurally identical CGCNNs independently predict FE, TMM, and BG for pre‐screening the potential AFMs. GA iteratively optimizes latent vectors (*Z*) for targeted generation. DFT is employed to relax crystal structures and verify AFM stability.

Our AFM discovery framework integrates four domains: structure generation via CDVAE‐DA, property‐based pre‐screening using CGCNNs, targeted optimization of latent vectors (*Z*) through GA, and DFT validation, performing structural relaxation and AFM stability assessment, as schematized in **Figure** **1**. This approach enables us to generate and identify new AFMs in a vast unexplored compositional space. First, we utilize the CDVAE‐DA to produce high‐quality and reasonable novel crystal structures. Here, the input crystal structure is denoted as *M*(*A*, *X*, *L*), where *A* represents atom types, *L* is the periodic lattice vectors defining the periodic symmetry, and *X* denotes atomic coordinates within the unit cell (details see Section S1, Supporting Information). We implement DA by applying affine rotations R to reorient lattice vectors and coordinates of input crystal structures into specific orientations per training batch. Meanwhile, we employ transfer learning by fine‐tuning the MP‐20‐pretrained CDVAE‐DA on the limited AFM‐specific dataset^[^ ^28^ ^]^ (696 materials with ≤ 20 atoms per unit cell) to enable targeted generation of novel structures from latent space vectors. Second, three structurally identical CGCNNs with distinct weights independently predict FE, TMM, and BG for pre‐screening the potential AFMs. Third, GA are used to update the latent vectors by crossing over the representations of generated crystals, preserving representations encoding AFM characteristics. Finally, DFT is used for crystal relaxation and property calculations of the generated crystals to confirm the new AFMs.

### 2.2 CGCNN Structure and Property Dataset Construction

We develop three structurally identical CGCNNs to predict FE, TMM, and BG, respectively (see Figure S1, Supporting Information). The graph representation encodes atomic nodes through feature vectors combining one‐hot encoded elemental properties (atomic mass, polarizability, covalent radius).^[^ ^24^, ^25^ ^]^ Edge attributes capture interatomic interactions using exponentially decayed distance filters between neighboring atoms within a 6 Å cutoff radius. From the Materials Project database,^[^ ^28^ ^]^ we extract 19174 magnetic materials and their corresponding properties. For FE training, we use the entire dataset. For TMM training, we focus on data within 0–10 *μ*_B_ f.u.^−1^ (amounting to 13418 structures) to reduce the impact of limited data above 10 *μ*_B_ f.u.^−1^. For BG training, we strategically downsample the dominant zero‐gap subset (10708 materials) by 80%, generating a balanced subset of 10607 materials with controlled phase representation. The data distribution of FE, BG, and TMM is shown in Figure S2 (Supporting Information).

## 3 Result

### 3.1 Training Results of CDVAE‐DA

**Figure 2:**
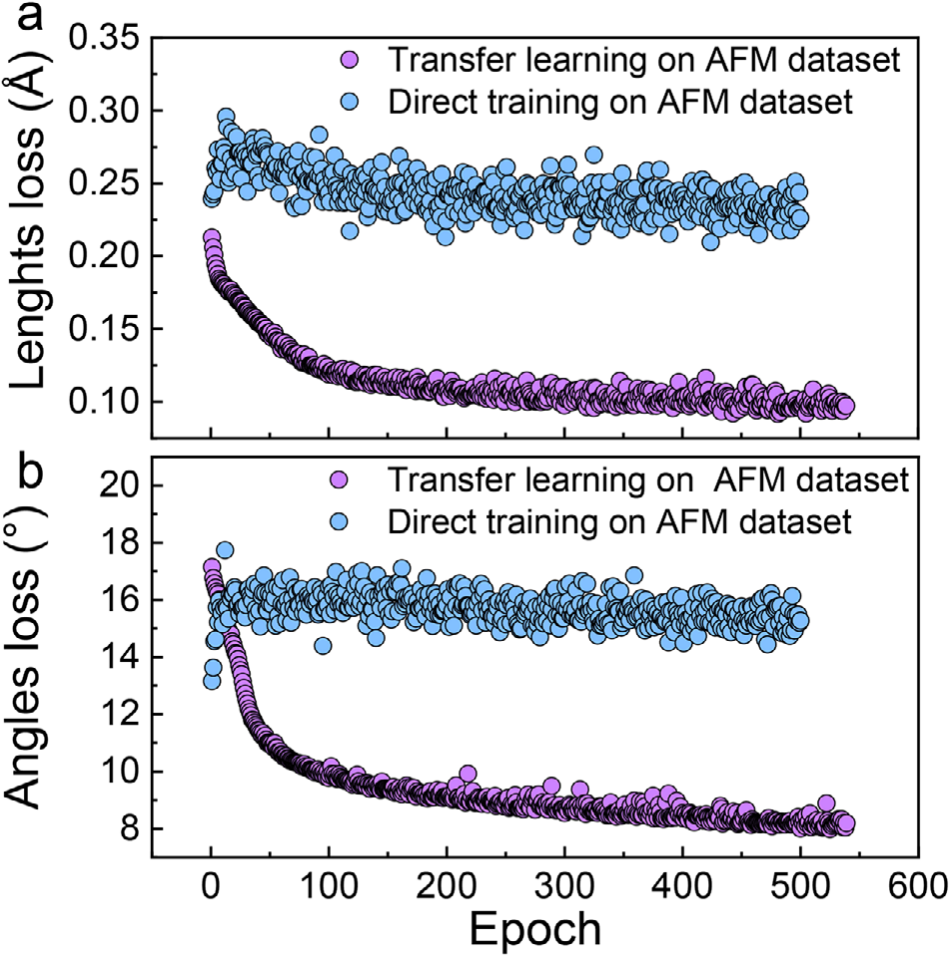
Training performance of CDVAE‐DA for lattice parameter prediction. a) MAE loss for the lengths of lattice vectors. b) MAE loss for inter‐axial angles. Blue points represent direct training on the AFM dataset. Purple points correspond to transfer learning (pretraining on the MP‐20 dataset followed by fine‐tuning on the AFM dataset).

To more intuitively reflect the prediction error, the mean absolute error $\mathrm{MAE}\;=\frac{1}{n}\;\sum_{i=1}^{n}\left|{\hat{y}}_{i}-y_{i}\right|$ is adopted to evaluate the CDVAE‐DA, where *y_i_* and ${\hat{y}}_{i}$ represent the real data and predicted data, *n* denotes the total number of samples. The initial training of CDVAE‐DA using the AFM dataset (696 materials with ≤ 20 atoms per unit cell) reveals fundamental challenges in network convergence. As depicted in **Figure** **2**a,b (blue traces), the oscillating losses failing to converge for the lattice vector lengths and inter‐axial angles during progressive epochs indicate the network's failure to effectively learn the structural distribution of AFM materials. This limitation stems from two intrinsic characteristics of the AFM dataset: its restricted sample size compared to the MP‐20 dataset (45231 structures),^[^ ^28^ ^]^ and its broader elemental diversity, complicating feature extraction.

To address these challenges, we implement a two‐stage transfer learning strategy^[^ ^22^, ^29^ ^]^ (see Figure S3, Supporting Information). First, the CDVAE‐DA is trained on the MP‐20 dataset^[^ ^28^ ^]^ to establish foundational knowledge of crystal chemistry, including lattice symmetry patterns and atomic coordination preferences. We also benchmark the reconstruction and generation capabilities of CDVAE‐DA against FTCP,^[^ ^30^ ^]^ CDVAE,^[^ ^16^ ^]^ and DiffCSP.^[^ ^31^ ^]^ Across these methods, CDVAE‐DA attains the highest compositional validity at 90.68%, demonstrating a relative advantage over alternative approaches in generating chemically plausible crystal structures (see Section S5, Supporting Information). Subsequently, the pre‐trained CDVAE‐DA is fine‐tuned on the AFM dataset, enabling targeted learning of magnetic structure correlations while preserving general crystallographic principles. As evidenced by the red traces in **Figure** **2**a,b, this hierarchical approach achieves stable convergence with monotonically decreasing prediction errors. After 500 epochs, the inter‐axial angle deviation is reduced by ≈50% (from ≈16° to ≈8°), and the lattice constant error is decreased by ≈60% (from ≈0.25 to ≈0.10 Å) compared to direct AFM training. These metrics confirm the framework's enhanced capability to capture both geometric and magnetic characteristics of AFM systems.

### 3.2 Performance of CGCNNs

**Figure 3:**
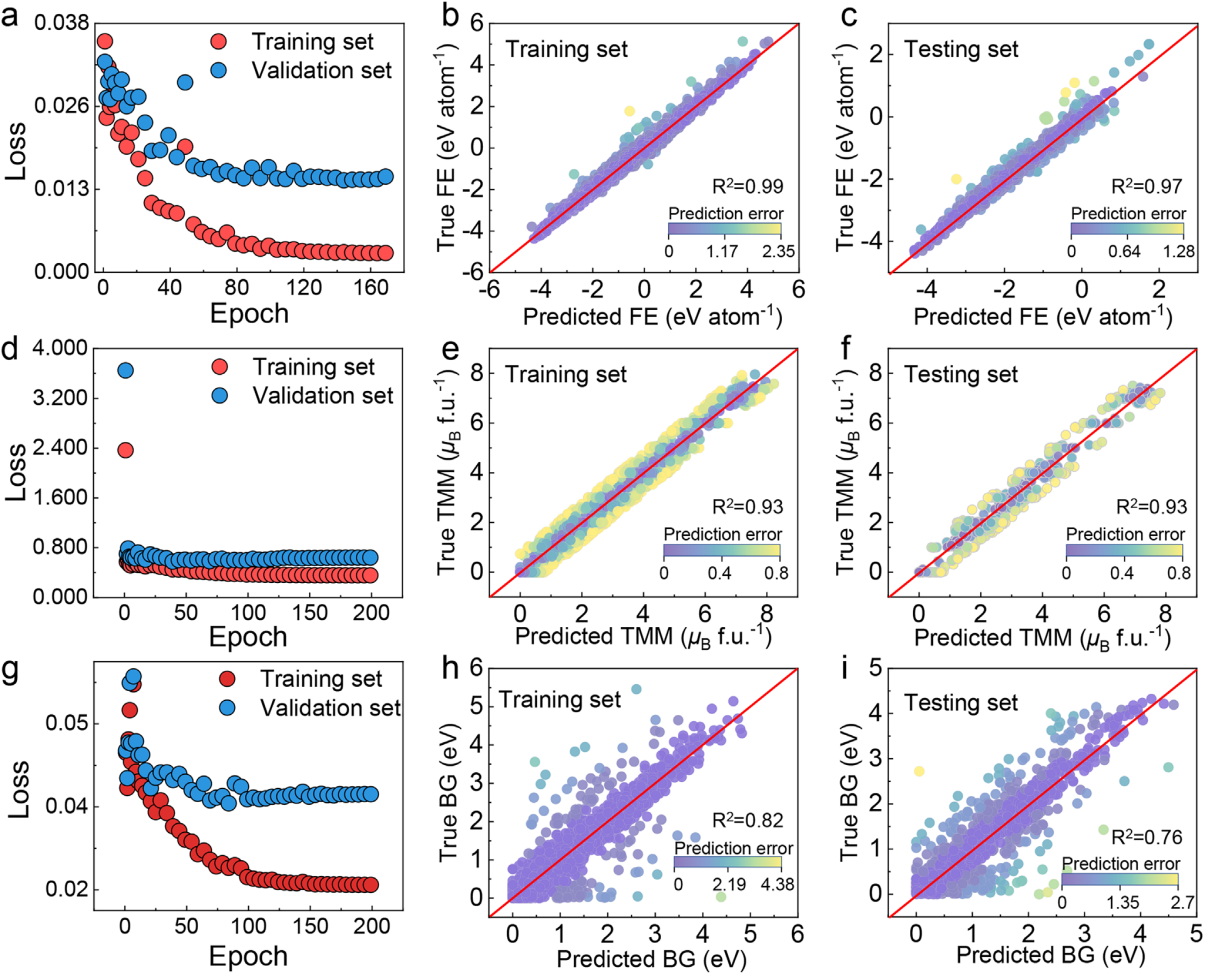
Performance evaluation of CGCNNs in predicting the different physical properties. Learning curve of CGCNNs showing training and validation loss as a function of epochs in predicting a) FE, d) TMM, and g) BG. Comparison between the true and predicted b,c) FE, e,f) TMM, and h,i) BG on the training and testing set.

During the CGCNN training, the mean squared error (MSE) is employed as the loss function. **Figure** **3** illustrates the performance of CGCNNs in predicting three key material properties: FE, TMM, and BG. For FE prediction, the CGCNN achieves low training and validation losses of 0.0029 and 0.0147, respectively (Figure 3a). This indicates the CGCNN's ability to effectively capture the relationship between crystal structures and FEs. The scatter plots for both training (Figure 3b) and testing (Figure 3c) sets show data points closely aligned along the diagonal, further demonstrating the CGCNN's high prediction accuracy.

For TMM prediction, the CGCNN attains training and validation losses of 0.3568 and 0.576 (Figure 3d). The data in the training (Figure 3e) and testing (Figure 3f) sets also show a close alignment along the diagonal, highlighting the CGCNN's effectiveness in identifying TMM for diverse crystal structures.

In the case of BG prediction, the CGCNN's training and validation losses are 0.024 and 0.037, respectively (Figure 3g). This indicates its capability to map crystal structures to BGs. Figure 3h reveals that the predicted BGs mainly cluster in the 0–3 eV range, with a dense concentration near the diagonal. The testing set data (Figure 3i) also primarily fall within the 0–3 eV range and align closely with the diagonal, suggesting good generalization ability. However, for crystal structures with BGs exceeding 5 eV, there is a slight deviation between predicted and actual values. This discrepancy likely arises from the limited number of such large‐BG structures in the training data, which may have restricted the CGCNN's ability to learn their specific features.

### 3.3 Crystal Structure Generation with GA

In our framework, three CGCNNs predict FE, TMM, and BG for the generated structures. These properties are used to determine the fitness score in the GA. Since stable AFMs typically exhibit negative FE and lower TMM, and considering the error on the test set of CGCNN networks, we set the fitness criteria for GA as FE < 0 eV atom^−1^ and TMM within 0–0.7 *μ*_B_ f.u.^−1^. In addition, given the significant potential of both AFM metals and semiconductors for spintronic applications, a BG criterion spanning 0–1 eV is adopted to cover both types of materials. To enable AFM ordering, which requires even‐numbered magnetic atoms in spin sublattices, we preferentially select structures with even magnetic atom counts during each GA generation, assigning elevated fitness scores accordingly. Subsequently, structures undergo probabilistic selection based on fitness scores to initialize latent vectors for the next iteration of structure generations. Each step generates 100 structures for a total of 20 steps. The fitness settings and GA details are provided in Section S6 (Supporting Information).

**Figure 4:**
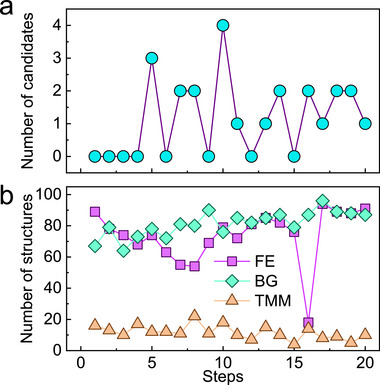
Candidate screening results during crystal structure generation. a) Number of AFM candidates at each step of the optimization process using GA. b) Number of structures satisfying the criteria of FE < 0 eV atom^−1^, TMM within 0–0.7 *μ*_B_ f.u.^−1^ and BG within 0–1 eV at each step.

As shown in **Figure** **4**a, AFM candidates yield peaks at four structures after 10 steps and approach stabilization after 15 steps. This trend may result from stochastic selection and swapping crossover points of the latent vectors in candidate structures, which preserves representations encoding AFM characteristics across successive iterations, steadily increasing the likelihood of producing AFM structures.

Between the 10th and 11th steps, the number of AFM candidates drops sharply (Figure 4a) while the number of structures meeting the BG criterion rises (Figure 4b). Since only a small fraction of the 100 final‐generation structures from the last step satisfy the BG criterion, the GA prioritizes latent vectors that encode low BG values at the expense of AFM feature retention for satisfying the BG criterion. This trade‐off reduces candidate counts while improving overall fitness. An analogous shift occurs between steps 15 and 16, when the algorithm favors BG and TMM targets, causing a transient fall in structures with FE < 0 eV atom^−1^. Nevertheless, 73.8% of all generated structures maintain FE < 0 eV atom^−1^, demonstrating good stability, and 23 potential AFM materials are screened (see Section S7, Supporting Information).

### 3.4 Identification of AFMs

DFT calculations are performed to determine the FEs of 23 candidate materials under both ferromagnetic (FM) and AFM spin configurations. The results reveal that MnS, OV_2_, FeO_4_P, and MnO exhibit lower FEs in the AFM configuration compared to their FM states. Using the Phase Diagram Analyzer from Pymatgen,^[^ ^32^ ^]^ the energy above the convex hull is calculated for the generated materials. Our calculations reveal that MnS, FePO_4_, and MnO exhibit an energy above the hull of 0 eV atom^−1^ (see Table S4, Supporting Information), which is indicative of their thermodynamic stability. Furthermore, to gain deeper insights into the magnetocrystalline anisotropy of these materials, we incorporate spin‐orbit coupling (SOC) effects and calculate the FEs of MnS, FePO_4_, and MnO with spins aligned along various crystallographic axes, as detailed in Table S5 (Supporting Information).

**Figure 5:**
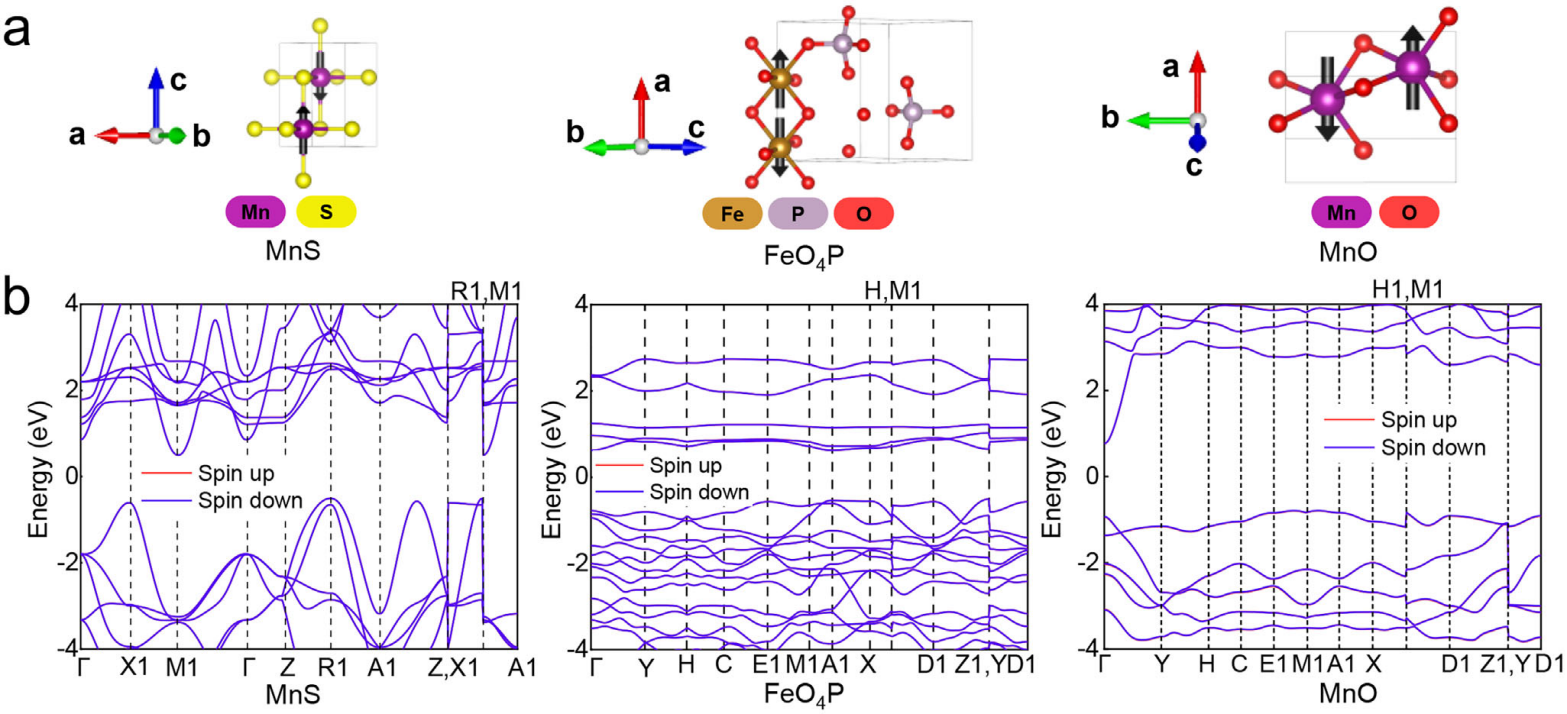
Crystal structures and electronic band structures of the three identified AFM semiconductors: MnS, FeO_4_P, and MnO. a) DFT‐relaxed crystal structures showing the AFM ground‐state spin configurations. Magnetic atoms (Mn, Fe) are highlighted, with spins aligned parallel to the easy axis and adjacent spins oriented antiparallel. b) Corresponding electronic band structures. The spin‐degenerate bands confirm the AFM ordering. The presence of BGs at the Fermi level (1.0076 eV for MnS, 1.1320 eV for FeO_4_P, and 1.5364 eV for MnO) indicates semiconducting behavior.

For MnS, the spin magnetic moments are predominantly located on the Mn atoms. Our SOC calculations reveal that the lowest FE is attained when the spins are aligned parallel to the *c*‐axis, identifying it as the easy axis of magnetocrystalline anisotropy in MnS. The DFT‐relaxed crystal structures and the AFM ground state of MnS are illustrated in **Figure** **5**a. The FE of MnS in the AFM ground state is calculated to be −27.1845 eV, which is lower than that in the FM state (−27.1611 eV). Figure 5b presents the corresponding band structure, exhibiting degenerate spin‐up and spin‐down channels, a hallmark of AFM order, along with a BG of 1.0076 eV near the Fermi level, indicative of semiconducting behavior. Similarly, FeO_4_P and MnO also adopt AFM ground states (see Table S6, Supporting Information). In FeO_4_P and MnO, the magnetic moments originate primarily from the Fe and Mn atoms, respectively, with the *a*‐axis identified as the easy axis of magnetocrystalline anisotropy in both structures (Table S5, Supporting Information). Their AFM ground‐state spin configurations are illustrated in Figure 5a. Band structure analyses (Figure 5b) confirm spin degeneracy without detectable spin splitting, consistent with AFM ordering. These systems also exhibit semiconducting electronic structures, with calculated BGs of 1.1320 eV for FeO_4_P and 1.5364 eV for MnO. Moreover, the generated MnS crystallizes in the tetragonal system, while FeO_4_P and MnO both adopt the monoclinic structure (Table S6, Supporting Information). These crystal systems differ from previously reported configurations and those documented in the Materials Project database.

Additionally, our DFT calculations identify AuMn_2_Pd, FNi, CMn, and IrSc as systems with FM ground states. Their crystal and electronic structures are provided in Section S7 (Supporting Information).

### 3.5 Crystal Structure Generation without GA

We further evaluate the generative performance of the framework without GA optimization. The trained CDVAE‐DA generates 5000 crystal structures spanning 84 elements, among which 3d transition metals (Fe, Mn, Cr, etc.) exhibit a high prevalence of 48.8% (Figure S5a, Supporting Information). This preferential incorporation of magnetic elements stems from the network's training on the AFM dataset, which may lead to the generation of structures with magnetic interactions. We subsequently employ the CGCNNs to predict the FE, TMM, and BG of these materials. The distributions of sample counts with respect to the TMM and BG values are shown in Figures S5b,c (Supporting Information), respectively. Here, we also focus on structures with FE < 0 eV atom^−1^, TMM between 0 and 0.7 *μ*_B_ f.u.^−1^, and BG between 0 and 1 eV. This screening yields 49 viable structures (Figure S5d and Table S7, Supporting Information). Among these, we identify 7 materials satisfying two additional constraints: an even number of magnetic atoms per unit cell (enabling AFM ordering) and total unit cell atom count < 20 (favoring structural simplicity).

**Figure 6:**
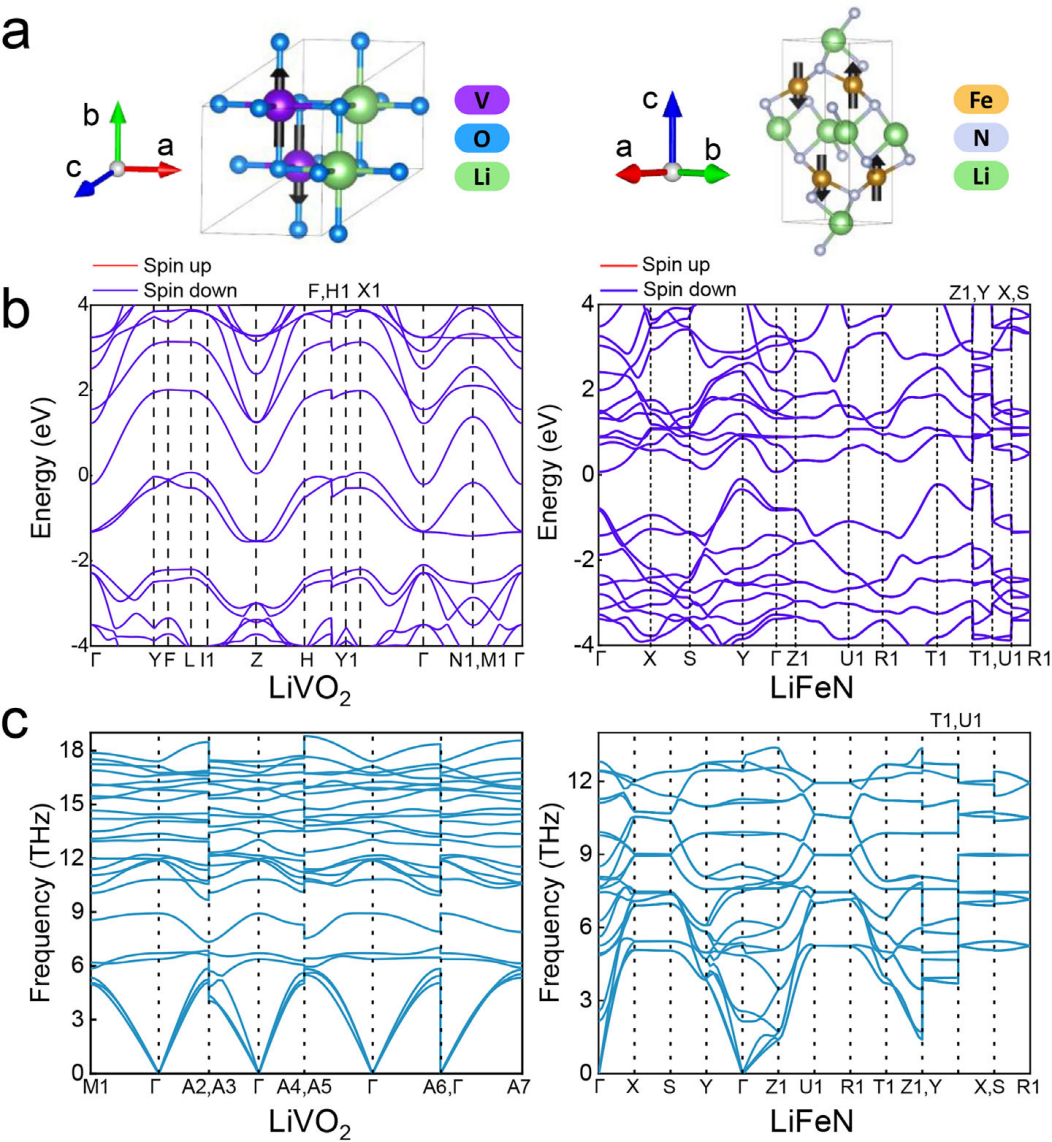
Crystal structures and electronic band structures of the two identified metallic AFMs: LiVO_2_ and LiFeN. a) DFT‐relaxed crystal structures showing the AFM ground‐state spin configuration. Magnetic atoms (V, Fe) are highlighted, with spins aligned parallel to the easy axis and adjacent spins oriented antiparallel. b) Corresponding electronic band structures. The absence of spin splitting confirms AFM ordering. Gapless band structures with degenerate bands crossing the Fermi level characterize the metallic behavior. c) Phonon dispersions of the LiVO_2_ and LiFeN.

Energy above hull and magnetic property analyses identify two stable metallic AFMs with negative FEs: LiVO_2_ and LiFeN (see Section S10, Supporting Information). In these compounds, the magnetic moments are primarily located on the V and Fe atoms, respectively. The DFT‐relaxed crystal structures of LiVO_2_ and LiFeN are shown in **Figure** **6**a. SOC calculations of magnetocrystalline anisotropy (Table S9, Supporting Information) indicate that the lowest‐energy state is achieved when the magnetic moments align along the *b*‐axis in LiVO_2_ and *c*‐axis in LiFeN. The AFM states exhibit energy lowerings of 0.5244 eV for LiVO_2_ and 1.4957 eV for LiFeN relative to their FM configurations (Table S10, Supporting Information). Their ground‐state AFM structure is shown in Figure 6a, featuring an antiparallel alignment of adjacent magnetic moments along the easy axis. Figure 6b displays the electronic band structures, where the absence of spin splitting demonstrates spin degeneracy consistent with AFM ordering. Moreover, they display gapless electronic structures, with spin‐degenerate band crossings at the Fermi level that confirm both metallic and AFM features. Phonon dispersion calculations show no imaginary frequencies, indicating that these materials are dynamically stable (Figure 6c). These results validate the coexistence of strong electron correlations and long‐range AFM order in the discovered compounds. During validation, four ferromagnetic and one non‐magnetic material are excluded (see Figure S6, Supporting Information for crystal and band structures).

## 4 Conclusion

We have developed a generation framework for AFM discovery. The CDVAE‐DA can achieve a 90.68% composition validity rate for generated materials. Guided by CGCNN predictions and GA with fitness criteria defined as FE <0 eV atom^−1^, BG within 0–1 eV, TMM within 0–0.7 *μ*_B_ f.u.^−1^, a total of 2000 structures over 20 steps are generated, and 23 candidate materials are screened. Through DFT validation, three semiconductor AFMs, MnS, FeO_4_P, and MnO, are confirmed. These materials exhibit stable AFM configurations, and their spin‐degenerate band structures align with AFM ordering. Additionally, we directly employ CDVAE‐DA and CGCNNs without GA for AFM design. Among 5000 generated structures, two stable metallic AFMs are identified: LiVO_2_ and LiFeN. The results demonstrate that GA can effectively modulate the distribution of latent vectors to accelerate AFM generation. Our work validates the effectiveness of the proposed material design framework in generating AFMs for next‐generation spintronics and provides a versatile template applicable to a broader range of material systems. Future work could incorporate constraints related to time‐reversal symmetry and spatial inversion symmetry to generate a wider range of stable, unique, and novel AFM materials.

## 5 Experimental Section

### Crystal Diffusion Variational Autoencoder

The CDVAE‐DA is an extension of the original CDAVE,^[^ ^16^ ^]^ employing SE(3)‐equivariant periodic graph neural networks (PGNNs) to preserve crystal symmetries and boundary interactions in the encoding and decoding processes individually. The encoder utilizes DimeNet++^[^ ^33^ ^]^ to map crystal structures *M*(*A*, *X*, *L*) to latent vectors *Z*, while the decoder combines a multilayer perceptron (MLP) with GemNet‐dQ^[^ ^34^ ^]^ for crystal structures reconstruction *M*′(*A*′, *X*′, *L*′). In the generated process, MLP networks are used to predict the number of atoms *N*, the lattice vectors *L*, and the composition *c* based on a *Z* sampled from normal distribution. Then, the structure $\tilde{M}\left(A_{0},X_{0},L\right)$ is randomly initialized using *N*, *L*, *c*, and *Z*. The decoder is utilized to perform annealed Langevin dynamics to denoise the coordinates *X* and atom types *A*, ultimately generating the new crystal structures (details see Section S1, Supporting Information).

### Data Augmentation

Generally, rotational operations effectively expand the training set, enabling data augmentation across the entire dataset.^[^ ^35^ ^]^ This is implemented by applying rotation matrices R to input structures *M*(*A*, *X*, *L*), yielding transformed crystals: RM(A,X,L)=M(A,RX,RL) with corresponding reconstructed outputs 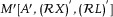. Here, R comprises six rotation matrices, which are used to rotate the coordinates *X* and lattice vectors *L*. Rather than applying all rotations per sample (6 × dataset expansion), one R per mini‐batch is randomly selected during each epoch. This balances augmentation benefits with computational efficiency. Consistent with established principles,^[^ ^36^, ^37^, ^38^ ^]^ training with transformed inputs induces output transformations that preserve equivariance. The CDVAE loss is therefore augmented with an equivariance term: 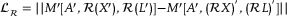. All other loss coefficients follow the CDVAE default settings.^[^ ^16^ ^]^ Main hyperparameters and the complete loss function for training CDVAE‐DA are shown in Section S1 (Supporting Information). All networks in this study are trained on servers equipped with RTX 4090 and A6000 GPUs.

### Transfer Learning Strategy

A transfer learning strategy is used to enhance the performance of the CDVAE‐DA network in generating AFMs by leveraging the correlation between the MP‐20^[^ ^28^ ^]^ dataset and the AFM dataset.^[^ ^28^ ^]^ The pre‐training and fine‐tuning methods are primarily adopted.^[^ ^22^, ^29^ ^]^ MP‐20 and AFM datasets are used as the source and target domains, respectively (see Figure S3, Supporting Information).

### Architecture of Graph Convolutional Neural Network

In CGCNN,^[^ ^24^, ^25^ ^]^ one‐hot encoding of the atomic masses, polarizabilities, and covalent radius corresponding to different atoms is performed to construct the node (3 × 64), and the interatomic distances between adjacent atoms are used as the edges. Subsequently, a fully connected network is set to integrate the atom features into a tensor (1 × 64). Two consecutive graph convolutional layers are then used to aggregate and pass messages, capturing the local neighborhood information and gradually building up the global features. Finally, based on a fully connected layer with an output channel of 1, all nodes and edges are aggregated to predict the properties, such as FE, TMM, and BG. The mean squared error $\mathrm{MSE}\;=\frac{1}{n}\;\sum {\left(\hat{y_{i}}-y_{i}\right)}^{2}$ is utilized to assess the error between the CGCNN output and the labels, where $\hat{y_{i}}$ is the predicted value and *y* is the true value, *n* denotes the total number of samples (details see Section S2, Supporting Information).

### Genetic Algorithm

GA is an optimization technique inspired by natural selection and genetic principles.^[^ ^26^, ^27^ ^]^ GA uses selection, crossover, and mutation to evolve a population of potential solutions, aiming to find the optimal or near‐optimal solution. In this study, the GA is used to optimize latent vectors, thereby promoting the generation of crystal structures with specified properties by CDVAE‐DA (details see Section S6, Supporting Information).

### First Principles Calculation

In the first‐principles calculations, Amsterdam Modeling Suite Quantum Espresso 7.1 (AMS‐QE) package^[^ ^39^ ^]^ is utilized to perform structural and geometric optimizations of the generated crystals, and AMS‐BAND (BAND 2025.102)^[^ ^40^ ^]^ is employed to calculate the electronic properties. In the AMS‐QE calculations, a Monkhorst–Pack *k*‐point mesh was employed for sampling the Brillouin zone. The number of *k*‐points along each lattice direction is chosen such that its product with the corresponding lattice constant (in Ångströms) ≈60, ensuring sufficient sampling accuracy. This *k*‐point grid is used in both structural relaxation and single‐point energy computations. A plane‐wave cutoff energy of 500 eV is applied to ensure convergence of the electronic structure calculations. During the structural optimization process, the lattice is constrained to prevent deformation. The energy convergence criterion is set to 1 × 10^−5^ Hartree. For calculations using AMS‐BAND, the Perdew‐Burke‐Ernzerhof (PBE) functional is employed to handle the generalized gradient approximation (GGA) of electronic exchange, along with a double‐zeta polarized (DZP) basis set of Slater‐type orbitals.^[^ ^41^ ^]^ The “unrestricted” option is selected to capture the spin polarization of electrons,^[^ ^42^ ^]^ and the frozen core level is set to “medium”. Spin‐orbit coupling (SOC) is included to evaluate magnetocrystalline anisotropy by calculating formation energies for spin configurations aligned along various crystallographic axes. To address the strong electron correlation in transition metal atoms, the GGA + *U* method is utilized for their *d* orbitals, incorporating specific Hubbard U values: 4.0 eV for Mn, 3.9 eV for Fe, 3.7 eV for Co, 6.2 eV for Ni, and 3.25 eV for V.^[^ ^28^ ^]^ Additionally, phonon dispersion relations of the generated crystals are computed using the AMS‐QE package. In phonon calculation, the *k*‐point mesh and interpolation are set to 9 × 9 × 9 and 100, respectively. The “Numerical” is selected to perform analytical phonon calculations, and the crystals are computed in 3 × 3 × 3 supercell based on their corresponding primitive cell.

## Conflict of Interest

The authors declare no conflict of interest.

## Author Contributions

Z.Z. conceived the idea and led the overall study. J.G., Z.Z., and Z.F. developed the Python code and performed DFT calculations. Z.Z. and J.G. analyzed the results and wrote the paper. J.G., Z.F., and H.F. produced the final figures and dataset clustering. Z.Z. and D.W. supervised the project. D.W. and H.W. discussed the data and commented on the manuscript.

## Supporting information



Supporting Information

## Data Availability

The data that support the findings of this study are available from the corresponding author upon reasonable request.
