# Supplementary material for: A Generative Framework for Predicting Antiferromagnets
Source: Adv Sci (Weinh). 2025 Sep 26;12(46):e09488. doi: 10.1002/advs.202509488 (PMC12697850; doi:10.1002/advs.202509488)
Supplement: Supplementary file 1 — Supporting Information [file ADVS-12-e09488-s001.pdf]

Supporting Information

A Generative Framework for Predicting Antiferromagnets

Jianhu Gong[1], Zhengming Zhang[1, *], Zhenyu Fan[1], Hanghang Fu[1], Hongchang Wang[1], Dunhui Wang[1, 2, *]

[1] *Zhejiang Provincial Key Laboratory of Data Storage, Hangzhou Dianzi University, Hangzhou, Zhejiang 310018, China*
[2] *National Laboratory of Solid State Microstructures, Nanjing University, Nanjing, Jiangsu 210093, China*

*Corresponding authors.
E-mails: zmzhang@hdu.edu.cn; wangdh@hdu.edu.cn

Section S1. Principle of CDVAE-DA

The CDVAE-DA extends the original CDVAE,[1] which learns the probability distribution of crystal structures and produces physically plausible configurations. A crystal structure is formally defined as a periodic arrangement of unit cells, where each unit cell M comprises three fundamental components: atomic species $A = (a_0, \dots, a_N) \in \mathbb{A}^N$, atomic coordinates $X = (x_0, \dots, x_N) \in \mathbb{R}^{N\times 3}$, and lattice vectors $L = (l_1, l_2, l_3) \in \mathbb{R}^{3\times 3}$. Here, N denotes the number of atoms in the unit cell, and $\mathbb{A}$ represents the set of all chemical elements. To effectively capture long-range interactions across periodic boundaries, the input crystal structure $M = (A, X, L)$ is processed through a multi-graph representation before being fed into the network.[1] The infinite periodic structure can be represented as[1]

$$\{(a_i', x_i' | a_i') = a_i, x_i' + k_1 l_1 + k_2 l_2 + k_3 l_3, k_1, k_2, k_3 \in \mathbb{Z}\} \qquad \text{S(1)}$$

where the integers k_1, k_2, k_3 translate the unit cell to tile the entire 3D space.

The CDVAE-DA employs an encoder to project high-dimensional crystal structures M into a low-dimensional latent space Z while preserving SE(3) invariance.[1] This encoding process establishes a probabilistic mapping $q(Z|M)$ that maintains the essential symmetry properties of crystalline materials. During decoding, latent variables sampled from Z are first transformed into noisy initial structures $\widetilde{M} = (\tilde{A}, \tilde{X}, L)$ via a multilayer perceptron. The core decoding component, a noise conditional score network (NCSN),[2] then iteratively refines these structures by estimating the score $\nabla_{\widetilde{M}} q_\sigma(\widetilde{M})$ under Gaussian noise σ. To generate samples from the model, an initial noise sample $\widetilde{M} = (\tilde{A}, \tilde{X}, L)$ is iteratively updated along the score direction via annealed Langevin dynamics to generate new structures[2]

$$\tilde{x}_i = \tilde{x}_{i-1} + \frac{\alpha_j}{2} s_\theta(\tilde{x}_{i-1}, \sigma_j) + \sqrt{\alpha_j} z_i \qquad \text{S(2)}$$

where $\tilde{x}_i$ represents the data distribution after the i-th denoising step, with $i = 1, 2, 3, \dots$. The adaptive step size at noise level σ_j is given by $\alpha_j = \epsilon \cdot \sigma_j^2 / \sigma_C^2$, where ϵ is the base step size, and $j = 1, 2, 3, \dots, C$ indexes the noise levels. $s_\theta(\tilde{x}_{i-1}, \sigma_j)$ is a conditional score network, and $z_i \sim \mathcal{N}(0, I)$ is Gaussian noise.[2] Through progressive denoising of atomic coordinates $\tilde{X}$ and atom types distribution $\tilde{A}$, the NCSN drives the system toward low-energy configurations that obey fundamental physical constraints.[2] A multilayer perceptron (MLP) is used

to predict the composition vector p_c. This prediction is then used to linearly perturb the atomic type distribution according to $\tilde{A} \sim (\frac{1}{1+\sigma_A} p_A + \frac{\sigma_A}{1+\sigma_A} p_c)$.[1] Here, p_A denotes the ground-truth atom type distribution, where $p_{A,ij} = 1$ if atom i corresponds to the true atom type j, and $p_{A,ij} = 0$ otherwise. Additionally, the atomic coordinates are perturbed by adding Gaussian noise $\tilde{X} \sim \mathcal{N}(X, \sigma_X^2 I)$.[1] A set of noise levels is defined for both atom types and coordinates, denoted as $\{\sigma_{A,j}\}_{j=1}^{C}$ and $\{\sigma_{X,j}\}_{j=1}^{C}$, respectively. The values are configured with $\sigma_{A,1} = 5$, $\sigma_{A,C} = 0.01$ for atom types and $\sigma_{X,1} = 10$, and $\sigma_{X,C} = 0.01$ for coordinates.[1]

Table S1. Main hyperparameters during CDVAE-DA training.

Hyperparameters	Values
Cost natom (λ_N)	1
Cost coord (λ_X)	10
Cost type	1
Cost lattice (λ_L)	10
Cost composition (λ_c)	1
Cost edge	10
γ	1
Beta (β)	0.01
Teacher forcing lattice	true
Sigma begin ($\sigma_{X,1}$)	10
Sigma end ($\sigma_{X,C}$)	0.01
Type sigma begin ($\sigma_{A,1}$)	5
Type sigma end ($\sigma_{A,C}$)	0.01
Number of noise level	50

In CDVAE-DA, rotation matrices $\mathcal{R}$ are employed to augment the training dataset. The set consists of six distinct rotation matrices:

$$\mathcal{R}_1 = \begin{bmatrix} 1 & 0 & 0 \\ 0 & 1 & 0 \\ 0 & 0 & 1 \end{bmatrix} \mathcal{R}_2 = \begin{bmatrix} 1 & 0 & 0 \\ 0 & 0 & 1 \\ 0 & 1 & 0 \end{bmatrix} \mathcal{R}_3 = \begin{bmatrix} 0 & 1 & 0 \\ 1 & 0 & 0 \\ 0 & 0 & 1 \end{bmatrix} \mathcal{R}_4 = \begin{bmatrix} 0 & 1 & 0 \\ 0 & 0 & 1 \\ 1 & 0 & 0 \end{bmatrix}$$

$$\mathcal{R}_5 = \begin{bmatrix} 0 & 0 & 1 \\ 1 & 0 & 0 \\ 0 & 1 & 0 \end{bmatrix} \mathcal{R}_6 = \begin{bmatrix} 0 & 0 & 1 \\ 0 & 1 & 0 \\ 1 & 0 & 0 \end{bmatrix} \qquad \text{S(3)}$$

These matrices are applied to rotate both the atomic coordinates X and lattice vectors $L = (l_1, l_2, l_3)^T$. For example, $\mathcal{R}_2$ corresponds to a 90° clockwise rotation around the x-axis, such that: $\mathcal{R}_2(l_1, l_2, l_3)^T = (l_1, l_3, l_2)^T$. An equivariance term

$\mathcal{L}_{\mathcal{R}} = ||M'[A', \mathcal{R}(X'), \mathcal{R}(L')] - M'[A', (\mathcal{R}X)', (\mathcal{R}L)']||$ is added into the original CDVAE[1] loss. $M'[A', \mathcal{R}(X'), \mathcal{R}(L')]$ represents the result of rotating the reconstructed output of input structure $M(A, X, L)$. $M'[A', (\mathcal{R}X)', (\mathcal{R}L)']$ is the reconstructed output of rotated structure $M(A, \mathcal{R}X, \mathcal{R}L)$. The total loss can be written as

$$\mathcal{L} = \mathcal{L}_{CDVAE} + \gamma\mathcal{L}_{\mathcal{R}} = \lambda_c\mathcal{L}_c + \lambda_L\mathcal{L}_L + \lambda_N\mathcal{L}_N + \lambda_X\mathcal{L}_X + \lambda_A\mathcal{L}_A + \beta\mathcal{L}_{KL} + \gamma\mathcal{L}_{\mathcal{R}} \quad \text{S(4)}$$

where $\mathcal{L}_c$, $\mathcal{L}_L$, $\mathcal{L}_N$, $\mathcal{L}_X$, and $\mathcal{L}_A$ denote the losses of composition, lattice vectors, number of atoms, atom coordinates and atom types between true and generated structures, respectively.[1] $\mathcal{L}_{KL}$ represents the Kullback–Leibler divergence. λ_c, λ_L, λ_N, λ_X, λ_A, β and γ are the weight coefficients of these losses, respectively. We follow the loss coefficients and noise levels in CDVAE[1] and set $\gamma = 1$. All models are trained with a batch size of 128.

Section S2. CGCNN structure

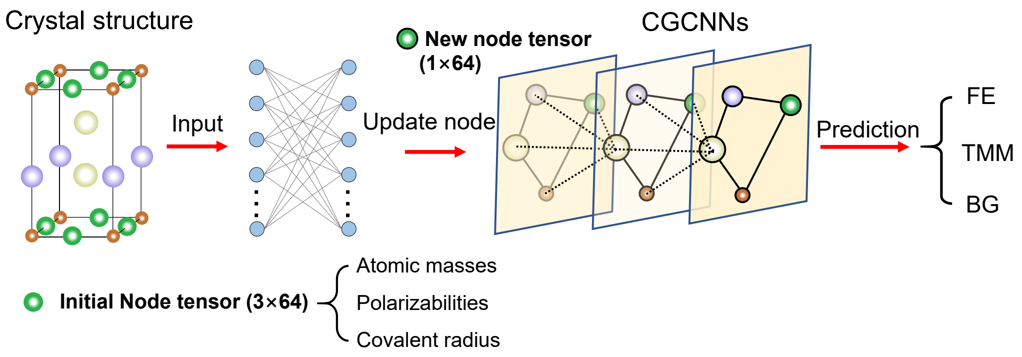

Figure. S1. Architecture of the CGCNN. Three structurally identical CGCNNs independently predict FE, TMM, and BG for pre-screening the potential AFMs.

Three structurally identical CGCNNs[3, 4] with distinct weights independently predict FE, TMM, and BG. In each CGCNN, the crystal graph data's nodes and edges are constructed based on different atoms and their interatomic distances with adjacent atoms. The node tensor (3×64) consists of the one-hot encoding of atomic masses, polarizabilities, and covalent radii corresponding to the atoms. Then, the node tensor is mapped into a tensor (1×64) by a fully connected network. Two graph convolutional layers are applied to further combine the node features with the edge features, obtaining a new crystal graph. Finally, a fully connected layer with an output of 1 aggregates all nodes and edges on the new crystal graph to predict the target property.

Since the FE of the crystal is typically negative, the hyperbolic tangent function[5] ($\tanh(x) = \frac{e^x - e^{-x}}{e^x + e^{-x}}$) is used as the activation function in the fully connected network, where x is the input data. The TMM and BG are both positive values, so the rectified linear unit[6] ($\mathrm{ReLU}(x) = \max(0, x)$) is applied as the activation function. The Adam algorithm,[7] which has adaptive learning rate characteristics, is selected as the optimizer for the entire deep learning network. Additionally, the ExponentialLR[8] as a learning rate scheduler in PyTorch is used to dynamically adjust the learning rate of an optimizer during the training process. During the training of the CGCNN, we mainly adjust the cutoff radius r_{cut} and the

learning rate. The r_{cut} is set to 6, 8, and 10 Å. The learning rate l_r is set to 0.005, 0.01, 0.0005, and 0.001. The training epoch is 200. We use the grid search method to train the network under different combinations of r_{cut} and l_r, and find that the CGCNN achieve the lowest prediction error in FE and TMM prediction when $r_{cut} = 6$ Å and $l_r = 0.005$. For $r_{cut} = 6$ Å and $l_r = 0.001$, CGCNN exhibits the lowest error in BG prediction.

Section S3. Data distribution of FE, BG and TMM

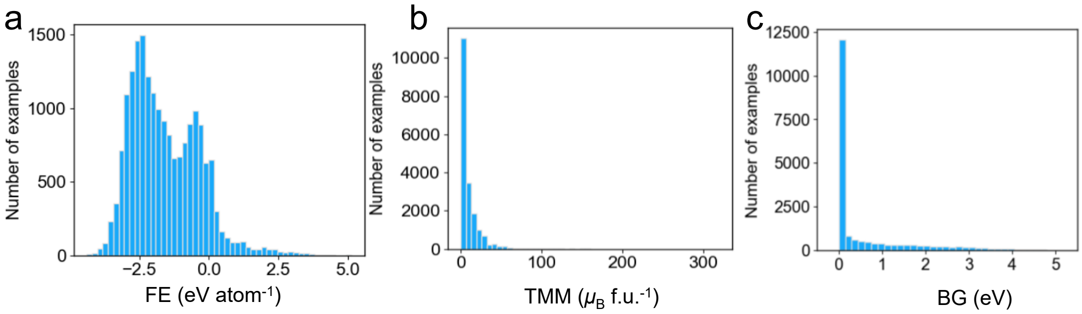

Figure. S2. Distribution of the dataset used for training the CGCNN

Using the Materials Project database,[9] 19,174 magnetic crystal structures and their corresponding properties are obtained to construct the dataset. Due to the different distribution of the FE, TMM, three identically structured CGCNNs are trained separately using three different preprocessing strategies. As can be seen from Figure S2a, the distribution of FE data is relatively uniform. Therefore, we use the entire dataset for CGCNN training in predicting FE. As shown in Figure S2(b-c), the distributions of TMM and BG are extremely uneven compared to that of FE. Therefore, we employ two data augmentation strategies to optimize the dataset. For the TMM, there are 13,418 structures within the 0-10 μ_B f.u.$^{-1}$. Since a few structures with values greater than 10 μ_B f.u.$^{-1}$ could degrade the network performance, we only use the data within 0-10 μ_B f.u.$^{-1}$ for training the CGCNN. Due to the predominance of metals with BG = 0, a data reduction technique is employed to address the severe class imbalance and optimize the performance of the CGCNN on the BG dataset. Specifically, 20% of the metallic samples (BG = 0 eV) are randomly selected and merged with all non-zero BG samples to form a balanced subset of 10,607 crystal structures. In the prediction of FE, TMM, and BG, we divide the dataset into training, validation, and testing sets in a 6:2:2 ratio.

Section S4. Transfer learning strategy

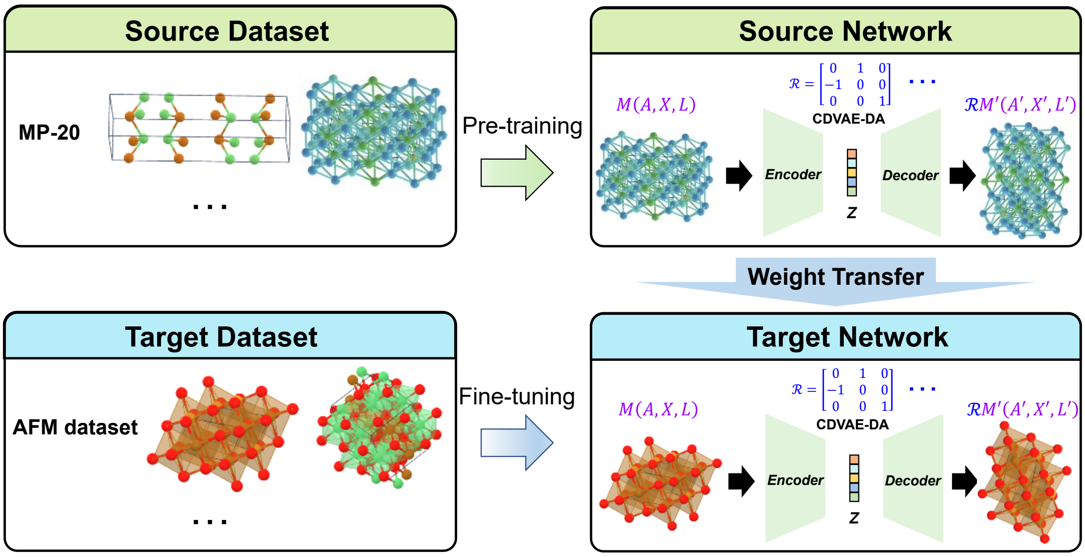

Figure. S3. Schematic illustration of the transfer learning framework. A source network is first trained using MP-20 dataset. Source network is transferred to the target network to initialize the parameters, and the AFM dataset is used to further optimize the parameters of target network.

The small size of the AFM dataset,[9] comprising only 696 structures with ≤ 20 atoms per unit cell, limits the generation precision of the CDVAE-DA. Based on a transfer learning method, we optimize the CDVAE-DA to generate AFMs by leveraging the correlation between the large MP-20[9] (45,231 structures) and the small AFM dataset.[9] Initially, we pre-train the CDVAE-DA using the MP-20 dataset as the source data, enabling the network to effectively generate crystal structures. Subsequently, the network is fine-tuned on the target AFM dataset to learn the characteristics of AFMs.

Section S5. Comparison of the performance of generative models

Table S2. Reconstruction and generation performance of generative model on the MP-20

Method	Match rate (%)	RMSE	Validity(%)		COV(%)	
			Struc.	Comp.	R.	P.
FTCP[10]	**69.89**	0.1593	1.55	48.37	4.72	0.09
CDVAE[1]	45.43	0.0356	100.0	86.70	99.15	99.49
DiffCSP[11]	–	–	100	83.25	**99.71**	**99.76**
CDVAE-DA	46.98	**0.0346**	**100.0**	**90.68**	98.89	95.0

We compare CDVAE-DA with the following three baselines, including the coordinate-based generation method FTCP[10], the CDVAE[1] and the DiffCSP[11] models. We compare the reconstruction and generation performance of these four models on the MP-20 dataset, with evaluation metrics including matching rate, root-mean-square error (RMSE), validity, Coverage-Recall (COV-R) and Coverage-Precision (COV-P).[1]

To calculate the matching rate and RMSE, we first use the generative network to reconstruct the corresponding crystal structures in the test set. The generated structures are screened using the StructureMatcher from Pymatgen,[12] with matching thresholds set to stol=0.5, angle_tol=10, and ltol=0.3. The matching rate represents the percentage of materials meeting the criteria out of the total generated structures. RMSE indicates the average difference in atomic distances over all matched materials and is normalized by $\sqrt[3]{V/N}$, where V represents the unit cell volume and N represents the total number of atoms.[1]

When testing the generative performance of the network, we mainly assess the validity of the generated materials and their similarity to real structural data. Structure validity[13] is defined as any pair of atoms having a shortest distance larger than 0.5 Å. Composition validity refers to the overall charge neutrality of the crystal structure, which can be calculated using the SMACT[14] library. Based on the evaluation method of CDVAE,[1] we also use Coverage-Recall (COV-R) and Coverage-Precision (COV-P) to assess the percentage of true materials being correctly predicted and the percentage of generated structures having high quality, respectively.

Section S6. Latent space optimization driven by genetic algorithm

In this study, the genetic algorithm[15, 16] (GA) is used to optimize latent vectors. Firstly, 100 latent vectors Z sampled from a normal distribution are fed into the CDVAE-DA to generate 100 crystal structures. The fitness of each individual within the GA is constructed based on the following criteria: FE<0 eV atom^{-1}, TMM between 0–0.7 μ_B f.u.$^{-1}$, BG between 0–1 eV and even number of magnetic atoms per unit cell. When a generated crystal structure meets one of the above criteria, the sample's fitness is assigned a value of 0.25. If a generated crystal structure satisfies all the criteria, its fitness is set to 1. The probability $P(i) = \frac{f(i)}{\sum_{j=1}^{N} f(j)}$ of selecting a crystal structure is calculated based on its fitness, where $f(i)$ represents the fitness of the i-th crystal structure, and N is the total number of crystal structures. Subsequently, the structures are selected according to the probability $P(i)$, and their latent vectors Z are subjected to random crossover to create new latent variables. Finally, based on these new latent vectors, the new crystals that inherit the characteristics of the previous generation crystals are reconstructed by CDVAE-DA.

Section S7. Properties and electronic structures of generated structures with GA optimization

Table S3. FEs, TMMs and BGs of 23 generated crystals predicted by the CGCNN.

Formula	FE (eV atom^{-1})	BG (eV)	TMM (μ_B f.u.$^{-1}$)
$Br_3Cs_2F_4Mn_2O_7TaTe$	−1.23196739	0.17632687	0.43414406
$BrMn_4Te_3ZnZr$	−1.16337105	0.65854627	0
NiF	−1.29424674	0.42728889	0.60056177
OV_2	−0.67032504	0.37670326	0.29780138
LiV_2F_7	−1.81287026	0.35483947	0.59888347
FeS	−1.37965506	0.31583688	0.67168246
$CuFe_2O_6$	−2.24730729	0.57888418	0.54706224
O_6TmV_2	−0.81780252	0.19720729	0
$AuNd_2O_7Sb_2V_2$	−3.02139829	0.86501831	0.61731877
$CsOV_2$	−1.68147054	0.74536484	0
OV	−2.02083737	0.9683223	0.55104945
CuV	−1.59312367	0.05682942	0.46249921
$HKO_{10}PV_2$	−2.29399597	0.56816864	0
MnS	−1.02362054	0.53003561	0.47200937
$SrFeO_3$	−1.18560716	0.58171982	0.65520315
$FePO_2$	−0.45621029	0.99781799	0.49218762
Co_2SiO_4	−1.03634238	0.59927821	0.21022177
$Cr_2CsCuMo_2O_5Te_5U_4$	−2.01762213	0.40753922	0.37677932
$AuMn_2Pd$	−3.01254173	0.33194262	0.08801696
Fe_2O_3	−2.50969820	0.82720214	0.36327119
IrSc	−1.10858246	0.45105544	0
$Cl_5Cs_2Mn_2O_6Te_4Tm$	−2.41142046	0.99551296	0.65962110
MnC	−1.57136861	0.52757251	0.65712340

Table S4. Energy above the hulls of the generated structures with GA.

Formula	Magnetic ordering	Energy above the hull (eV atom^{-1})
FeO_4P	AFM	0
OV_2	AFM	2.56804
MnO	AFM	0
MnS	AFM	0
FNi	FM	0
MnC	FM	0
IrSc	FM	0
$AuMn_2Pd$	FM	1.879143

Table S5. FEs (eV) with SOC for different magnetic orientations along the crystallographic axes in FeO_4P, MnO, and MnS.

Formula	*a*-axis	*b*-axis	*c*-axis	Easy axis
FeO_4P ($Fe_2O_8P_2$)	**−77.5392**	−76.9730	−76.4916	*a*
MnO (Mn_2O_2)	**−23.0529**	−23.0502	−22.8889	*a*
MnS (Mn_2S_2)	−18.0615	−18.0668	**−18.5511**	*c*

Table S6. FEs of the FM and AFM states and crystal systems of the optimized structures.

Formula	FM state (eV)	AFM state (eV)	Crystal system
FeO_4P ($Fe_2O_8P_2$)	−80.9764	−81.1939	Monoclinic
OV_2	−19.2041	−19.6359	Triclinic
MnO (Mn_2O_2)	−31.2748	−31.5213	Monoclinic
MnS (Mn_2S_2)	−27.1611	−27.1845	Tetragonal
FNi (F_2Ni_2)	−16.3791	−14.9945	Triclinic
MnC (Mn_2C_2)	−29.5622	−29.3878	Triclinic
IrSc (Ir_2Sc_2)	−21.8183	−21.8177	Triclinic
$AuMn_2Pd$	−19.8311	−19.7046	Triclinic

By including SOC, the FEs of FeO_4P ($Fe_2O_8P_2$), MnO (Mn_2O_2), and MnS (Mn_2S_2) with spins oriented along different crystallographic axes are calculated, as summarized in Table S5. The lowest energy occurs when spins in FeO_4P and MnO are aligned parallel to the *a*-axis, identifying it as the magnetocrystalline easy axis. In contrast, the easy axis in MnS is found to be along the *c*-axis. The ferromagnetic (FM) and AFM states of the eight generated structures are calculated by DFT, as summarized in Table S6.

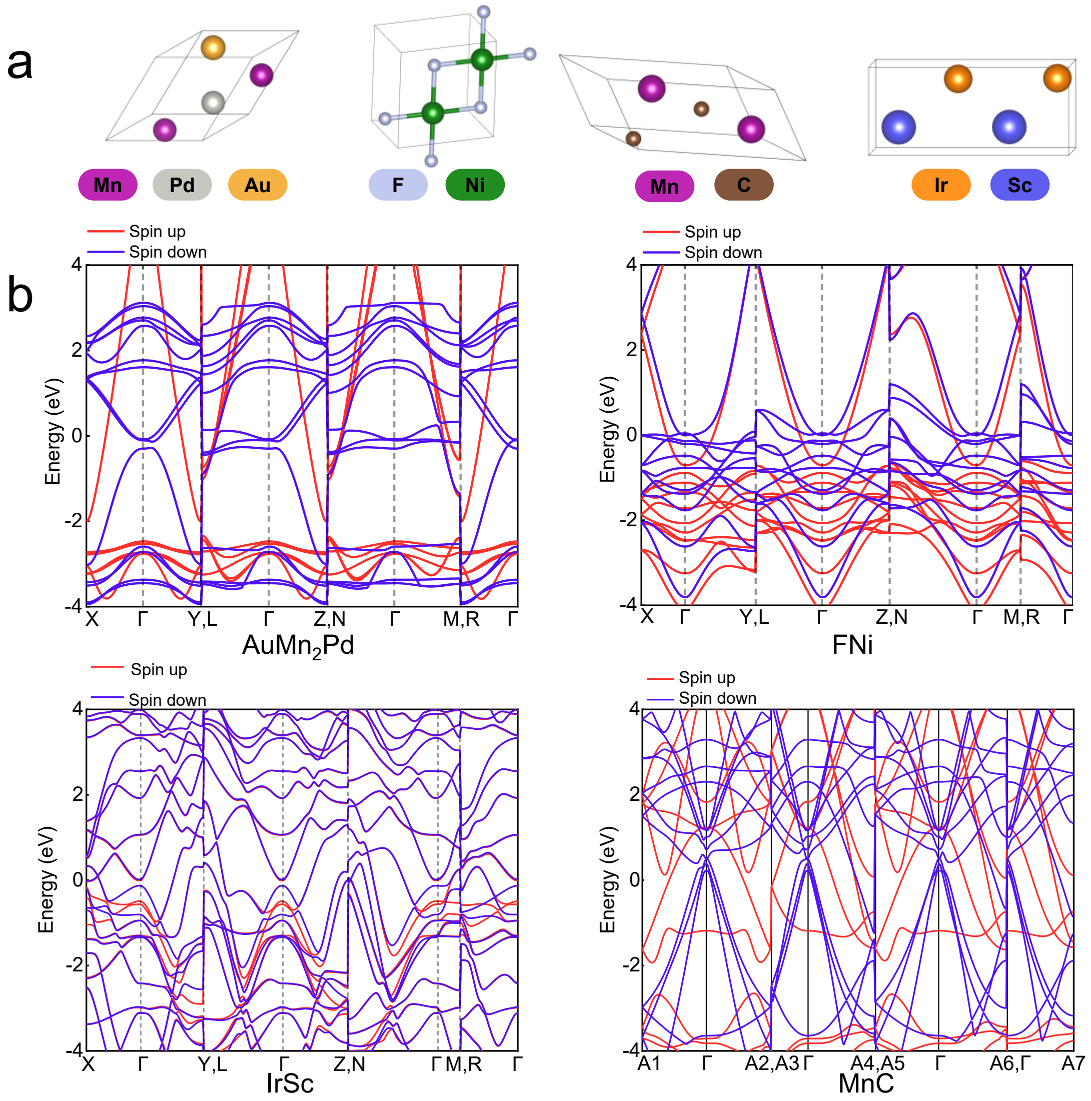

Figure S4. Crystal structures and electronic band structures of the four identified FMs: $AuMn_2Pd$, FNi, IrSc and MnC. (a) DFT-relaxed crystal structures. (b) Corresponding electronic band structures.

Section S8. Property distributions and novelty evaluation for structures generated by CDAVE-DA without GA optimization

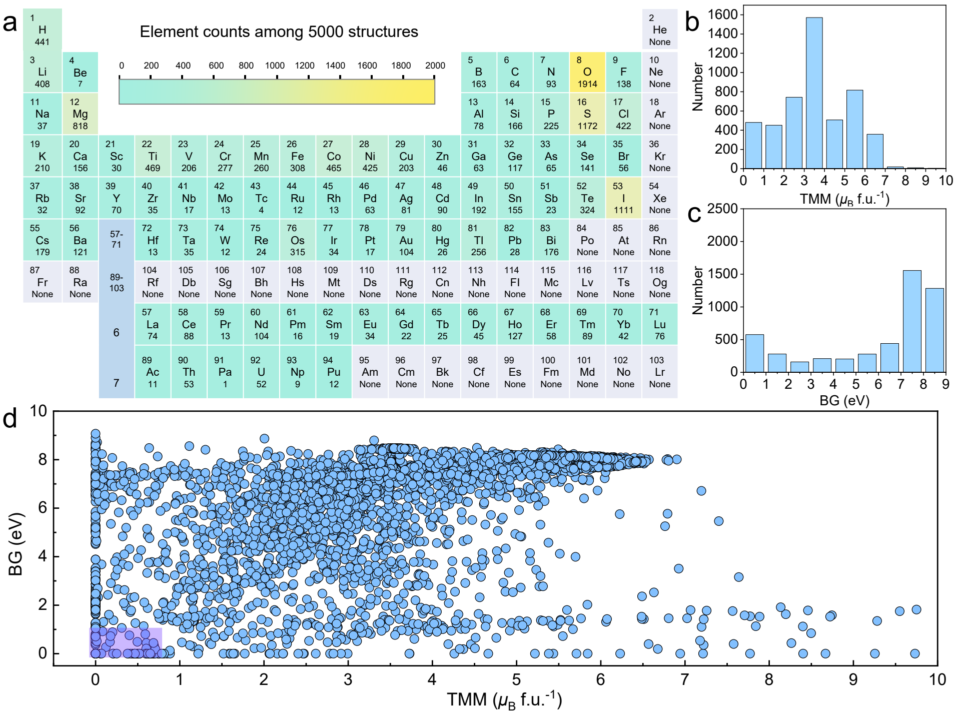

Figure S5. Structure generation and potential AFMs screening. (a) Preferences of element across 5,000 generated structures, with increasing orange intensity indicating the greater quantity of element. (b) Distributions of sample counts with TMM. (c) Distribution of BGs for the generated samples. (d)Under FE < 0 eV atom^{-1}, property distributions of generated structure. 49 potential AFMs with FE < 0 eV atom^{-1}, TMM between 0 and 0.7 μ_B f.u.$^{-1}$, and BG between 0 and 1 eV (see the purple area).

Figure S5 shows the element and property distributions of the generated structures without GA. We also perform a novelty test on the generated structures. Generally, novelty is defined as the percentage of the total generated structures that differ from the training data[17]. For the novelty assessment, we use the StructureMatcher[12] tool with specific tolerance parameters: ltol = 0.2, stol = 0.3, and angle_tol = 5°. Here, ltol refers to the tolerance for fractional length differences between structures, stol denotes the deviation of the average free length fraction $\sqrt[3]{V/N}$ between two structures, and angle_tol refers to the tolerance for angular differences. Using these settings, we identify a novelty rate of 99% on the AFM dataset, confirming the model's ability to explore new chemical spaces.

Section S9. FE, TMM and BG predictions for the structures generated by CDAVE-DA without GA optimization

Table S7. FEs, TMMs and BGs of 49 crystal structures predicted by the CGCNN.

**Formula**	FE (eV atom^{-1})	BG (eV)	TMM (μ_B f.u.$^{-1}$)
DyLiNaTe	−1.21462725	0.48854889	0.49562711
$CoTi_{13}$	−4.09115475	0.45935463	0
$CdErLa_2Sr_5Tl$	−0.46126572	0.91983247	0.08240589
$Mg_{11}Sc$	−0.04541709	0	0.43758067
$FeLi_5Ti_8$	−0.35104884	0.26682785	0.12222315
$CoLuMg_8$	−0.55844277	0.44361228	0.60390033
Li_3Ti_{11}	−2.21963542	0	0
$CHoLaPSi_2$	−0.72931797	0.32749971	0.69423246
$AlNi_2Ti_{11}$	−1.28013352	0.67593851	0
$LiTi_9$	−5.12272835	0	0
$CsMg_3$	−0.31559010	0	0.05718372
AgCaCsMg	−0.20180073	0	0.21176961
Ca_2GaTh	−2.32465453	0.48626522	0.17027987
$NiTi_{13}$	−1.31776580	0.37249194	0
$Co_2Li_3Ti_{13}$	−1.18396987	0.51656178	0
$CoLiTi_8$	−5.08216404	0.71637811	0
$CaLi_2Ti_7$	−5.12879535	0.80802495	0
$CaLuMg_8$	−0.57270617	0.20920301	0.60164382
$LiTi_2$	−2.27910432	0	0
$CoTi_9$	−4.78567669	0.70959358	0
$NiTi_9$	−4.77271969	0.66618723	0
$CaMg_8Tl$	−0.31104132	0	0.59846894
$CoTi_{19}$	−4.93926738	0.36798747	0
$CoLi_2Ti_7$	−5.12879533	0.80802495	0
Cs_3K	−0.16525074	0	0
CaCsLiMg	−0.13651968	0	0.46769988
Mg_9Tl	−0.64696870	0	0.37943642
$CoLi_3Ti_6$	−3.49591703	0.55235778	0
$LiMnTi_6$	−0.60824747	0.51017450	0.01509447
Ti_3Tl	−0.38265619	0	0.06937000
$LiTi_4$	−4.55151918	0	0
Co_2CrPTi_{10}	−0.67834033	0.72106179	0.17809937
Li_5Ti_9	−5.02961784	0	0
$MgTi_3$	−0.70072345	0	0.25011891
Co_2Ti_{12}	−1.22081909	0.70388237	0
$BeTi_9$	−4.99560962	0.76143071	0

Al_2LiTi_{11}	−1.64942547	0	0
AgLiMgNd	−0.60755585	0.44276127	0.68711708
$BiCs_2$	−0.125922831	0.377329	0.183256959
$CrMgS_4V$	−1.24850642	0.864077	0.60307612
FO_3V_2	−2.559513824	0.888664	0.683631434
$HfNi_3$	−0.388963694	0.129798	0.130828177
$Co_2Os_3S_5$	−0.207380433	0.883577	0.304434161
FeO_2	−1.02517154	0.793738	0.20868723
LiO_2V	−2.79387870	0.870647	0
MnO_4	−0.38854658	0.874959	0.26487806
LiFeN	−0.91990756	0.228117	0
Te_4TlV	−0.54804300	0.872796	0.56325004
$HMgNi_2$	−0.32739646	0.583601	0.42143167

We use the CGCNNs to separately predict the FEs, TMMs, and BGs of 5,000 crystal structures generated by CDAVE-DA without GA optimization. Among these, 49 crystal structures meeting the criteria of FE < 0 eV atom^{-1}, TMM between 0 and 0.7 μ_B f.u.$^{-1}$, and BG between 0 and 1 eV are screened out, as shown in Table S7. Furthermore, we identify 9 potential AFMs with even magnetic atom configurations and unit cells containing fewer than 20 atoms, which correspond to the last nine entries in Table S7.

Section S10. Magnetic properties and stability for the structures generated by CDAVE-DA without GA optimization

Table S8. Energy above the hulls and magnetic ordering for the generated structures without GA.

Formula	Magnetic ordering	Energy above the hull (eV atom^{-1})
LiFeN	AFM	0
$LiVO_2$	AFM	0
FeO_2	FM	0.556557
MnO_4	FM	0
$Co_2Os_3S_5$	FM	0
Te_4TlV	FM	0
$HfNi_3$	NM	0

Table S9. FEs (eV) with SOC for different magnetic orientations along the crystallographic axes in $LiVO_2$ and LiFeN.

Formula	*a*-axis	*b*-axis	*c*-axis	Easy axis
$LiVO_2$ ($Li_2V_2O_4$)	−57.1105	**−57.679**	−57.0876	*b*
LiFeN ($Li_4Fe_4N_4$)	−64.3893	−64.3899	**−66.2155**	*c*

Table S10. FE of FM and AFM states of $LiVO_2$ and LiFeN.

Formula	FM state (eV)	AFM state (eV)	Crystal system
$LiVO_2$ ($Li_2V_2O_4$)	−50.7839	−51.3083	Triclinic
LiFeN ($Li_4Fe_4N_4$)	−68.2272	−69.7229	Orthorhombic

Table S9 summarizes the FEs of the $LiVO_2$ ($Li_2V_2O_4$) and LiFeN ($Li_4Fe_4N_4$) for different spin orientations along selected crystallographic axes. $LiVO_2$ exhibits the minimum FE when the spins on the magnetic V atoms are aligned parallel to the *b*-axis, confirming this direction as the magnetocrystalline easy axis. Similarly, the *c*-axis is identified as the easy axis in LiFeN. Through DFT calculations, we obtain the FEs of $LiVO_2$ and LiFeN in both the FM and AFM ground states, as shown in Table S10. By comparison, it is found that both materials have lower FEs in the AFM ground state, indicating that they possess higher thermal stability in the AFM state.

Section S11. Crystal structures and electronic bands of five ferromagnetic and one non-magnetic materials.

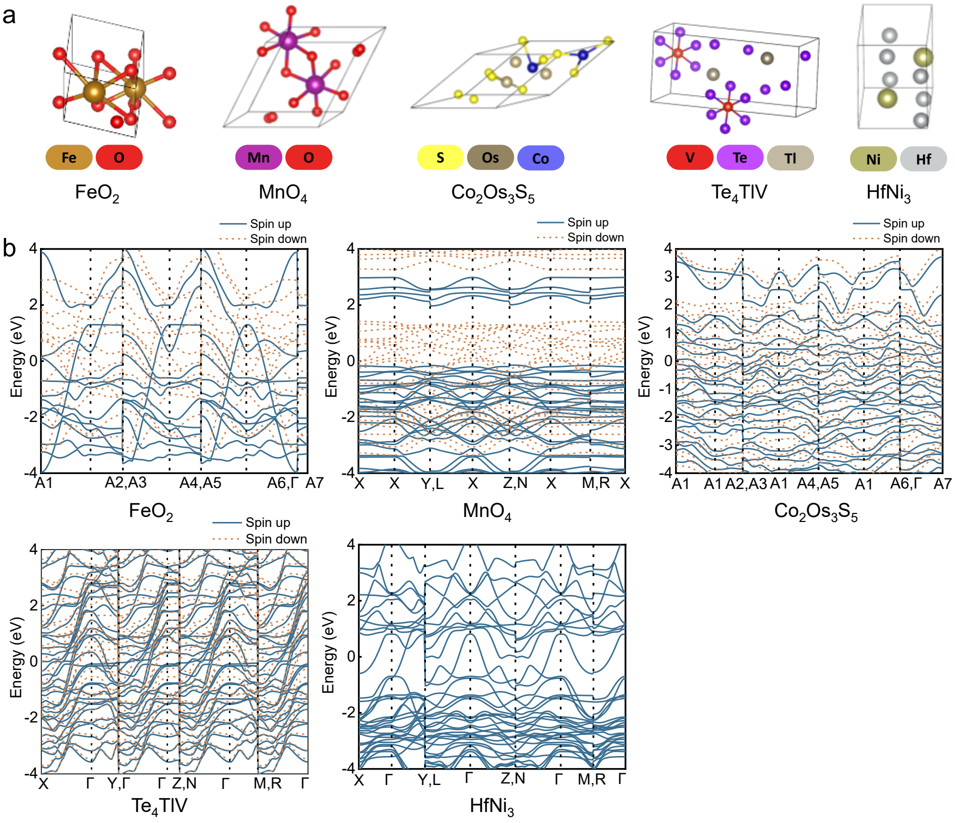

Figure S6. Crystal structures and electronic band structures of the five crystals. (a) FeO_2, MnO_4, $Co_2Os_3S_5$ and Te_4TlV showing the FM state. $HfNi_3$ exhibiting NM ground state. (b) Corresponding electronic band structures.

During the DFT calculations, four FM materials and one non-magnetic (NM) material are identified. Table S11 shows the FE of the magnetic states of these five materials. It is found that FeO_2 (Fe_2O_4), MnO_4 (Mn_2O_8), $Co_2Os_3S_5$ and Te_4TlV ($Te_8Tl_2V_2$) possess lower FEs in the FM state, showing a more stable thermodynamic state. Further, we calculate their electronic bands, which are shown in Figure S6. It can be clearly observed that the electronic bands of FeO_2, MnO_4, $Co_2Os_3S_5$ and Te_4TlV all reveal spin-splitting, with TMMs of 6.274, 4.0, 4.976 and 1.168 μ_B, respectively. Our calculations reveal that $HfNi_3$ (Hf_2Ni_6) exhibits a magnetic moment of zero, indicating a NM ground state, with a FE of –57.7263 eV. Additionally, the electronic band structures show that all these materials are metallic.

Table S11. FEs of the magnetic states in FeO_2, MnO_4, $Co_2Os_3S_5$, Te_4TlV and $HfNi_3$.

Formula	FM state (eV)	AFM state (eV)	NM state (eV)
FeO_2 (Fe_2O_4)	−20.9651	−20.8538	–
MnO_4 (Mn_2O_8)	−62.5629	−56.3560	–
$Co_2Os_3S_5$	−57.9752	−57.9314	–
Te_4TlV ($Te_8Tl_2V_2$)	−45.4560	−45.0468	–
$HfNi_3$ (Hf_2Ni_6)	–	–	−57.7263

References

[1] T. Xie, X. Fu, O.-E. Ganea, R. Barzilay, T. Jaakkola, *International Conference on Learning Representations* **2022**, https://doi.org/10.48550/arXiv.2110.06197.

[2] Y. Song, S. Ermon, *Advances in Neural Information Processing Systems 32* **2019**.

[3] T. Xie, J. C. Grossman, *Phys. Rev. Lett.* **2018**, *120*, 145301.

[4] N. T. Hung, R. Okabe, A. Chotrattanapituk, M. Li, *Adv. Mater.* **2024**, *36*, 2409175.

[5] S. R. Dubey, S. K. Singh, B. B. Chaudhuri, *Neurocomputing* **2022**, *503*, 92.

[6] H. Zhang, H. Nguyen, X.-N. Bui, B. Pradhan, P. G. Asteris, R. Costache, J. Aryal, *Eng. Comput.* **2022**, *38*, 3901.

[7] D. P. Kingma, J. Ba, *arXiv preprint arXiv: 1412* **2014**, *6980*.

[8] Z. Li, S. Arora, *arXiv preprint arXiv:.1910* **2019**, *07454*.

[9] A. Jain, S. P. Ong, G. Hautier, W. Chen, W. D. Richards, S. Dacek, S. Cholia, D. Gunter, D. Skinner, G. Ceder, K. A. Persson, *APL Mater.* **2013**, *1*, 011002.

[10] Z. Ren, S. I. P. Tian, J. Noh, F. Oviedo, G. Xing, J. Li, Q. Liang, R. Zhu, A. G. Aberle, S. Sun, X. Wang, Y. Liu, Q. Li, S. Jayavelu, K. Hippalgaonkar, Y. Jung, T. Buonassisi, *Matter* **2022**, *5*, 314.

[11] R. Jiao, W. Huang, P. Lin, et al., *Advances in Neural Information Processing Systems,* **2023**Curran Associates, Inc, New Orleans, LA, USA, **2023**.

[12] S. P. Ong, W. D. Richards, A. Jain, G. Hautier, M. Kocher, S. Cholia, D. Gunter, V. L. Chevrier, K. A. Persson, G. Ceder, *Comp. Mater. Sci.* **2013**, *68*, 314.

[13] C. J. Court, B. Yildirim, A. Jain, J. M. Cole, *J. Chem. Inf. Model.* **2020**, *60*, 4518.

[14] D. Davies, K. Butler, A. Jackson, J. Skelton, K. Morita, A. Walsh, *J. Open Source Softw.* **2019**, *4*, 1361.

[15] A. Lambora, K. Gupta, K. Chopra, in *Proceedings of the International Conference on Machine Learning, Big Data, Cloud and Parallel Computing (COMITCon)* **2019**, pp. 380-384.

[16] S. Katoch, S. S. Chauhan, V. Kumar, *Multimed. Tools Appl.* **2021**, *80*, 8091.

[17] C. Zeni, R. Pinsler, D. Zügner, A. Fowler, M. Horton, X. Fu, Z. Wang, A. Shysheya, J. Crabbé, S. Ueda, R. Sordillo, L. Sun, J. Smith, B. Nguyen, H. Schulz, S. Lewis, C.-W. Huang, Z. Lu, Y. Zhou, H. Yang, H. Hao, J. Li, C. Yang, W. Li, R. Tomioka, T. Xie, *Nature* **2025**, *639*, 624.
